# Systemic LPS resulted in a transient hippocampus malfunction but a prolonged corpus callosum injury

**DOI:** 10.1186/s12871-017-0396-1

**Published:** 2017-08-14

**Authors:** Jie Zhang, Aiyuan Li, Zongbin Song

**Affiliations:** 1 Department of Anesthesiology, The Maternal and Child Health Hospital of Hunan Province, Changsha, 410008 China; 2 Department of Anesthesiology, Xiangya Hospital, Central South University, Changsha, Hunan 410008 People’s Republic of China

**Keywords:** Lipopolysaccharide, Long term potentiation, Matured white matter injury, Axon compound action potential

## Abstract

**Background:**

To investigate the effect of systemic lipopolysaccharide (LPS) on function of hippocampus and corpus callosum (CC) in adult rats.

**Methods:**

Adult rats with mature white matter tract were divided into systemic LPS and saline injection groups. Animal were euthanized following 3 daily injections (day 3) and 3-day after cessation of injections (day 6). At both time points, hippocampal long term potentiation (LTP) and CC compound action potentials (CAP) were recorded, beta amyloid precursor protein (β-APP) level in CC tissue was measured by Western blot, and microglia activation was examined by immunostaining and proportional area analysis.

**Results:**

Systemic LPS significantly decreased amplitude of both post tetanic potentiation (PTP) and LTP at day 3, but PTP and LTP turned to be normal at day 6. CAP was significantly declined at day 3 but was further declined at day 6. The β-APP levels in CC tissues of LPS injected rats were significantly higher than that of saline group at both time-points. Interestingly, proportional area measurement disclosed that microglial areas in both hippocampus and CC significantly expanded at day3, but at the day 6, microglial area decreased in hippocampus but further increased in CC.

**Conclusion:**

Systemic LPS resulted in a transient hippocampus malfunction but a prolonged CC injury. Microglia activation may correlate with such LPS induced white matter injury.

## Background

Sepsis associated encephalopathy (SAE) is frequently observed in intensive care units and the extent of delirium is a predictor for severity of the SAE [1, 2]. The primary etiology of the SAE and associated delirium is not clear but it is known that endotoxin from invading pathogens, especially from gram-negative bacteria [3], plays a significant role in provoking brain injury [4, 5]. Hence, lipopolysaccharide (LPS) has been widely used in studying potential mechanisms of the SAE. As long-term potentiation (LTP) is thought to reflect hippocampal learning and memory functions [6], direct effects of LPS on LTP have been previously studied ex vivo, and inhibition of LTP had been reported [7, 8]. Some authors performed intracranial delivery of LPS for 4 weeks and observed reduction of LTP generation in the CA1 area [8]. However, systemic delivery of LPS to evoke inflammation and examine hippocampal functional LTP change is more relevant to real conditions of clinic endotoxemia and associated neuropsychiatry disorders. In addition, the endotoxin concentration in the brain during toxemia may be much lower than previously thought [9].

On the other hand, in a series of neuropathological studies of SAE by either postmortem observation or magnetic resonance imaging (MRI), the authors discovered marked white matter anomalies in both infants and older adults [10–12] and demonstrated a positive correlation between delirium duration and white matter integrity changes [13]. In animal studies, a line of research focused on fetal animals and demonstrated that maternal endotoxemia negatively affected white matter development [14, 15]. Yet little is known whether endotoxemia impacts mature white matter function in adult animals. Studying adults is important in light of the vast structural differences between fetal, new-born and mature white matter [16–19]. We attempt to address this question in the current work by using two well-established white matter injury tests: axons compound action potential (CAP) recording and analysis of β-amyloid precursor protein (β-APP) density in white matter tracts [20–23]. We used rat corpus callosum (CC) as representative white matter to perform our study. Microglia activation was also examined in both hippocampus and CC, as a number of researchers have reported associations of microglia activation with observed brain injuries [24, 25]. Further, proportional area has been reported to be a valuable parameter to evaluate microglia activation states [26]; therefore, proportional area was measured and analyzed to monitor corresponding microglial changes.

In addition, prolonged neuroinflammation following systemic inflammation has been reported, especially in rodents. For example, a group of authors reported that repeated systemic LPS injection evoked a pronounced prolonged neuroinflammation, as indicated by gene expression of and positive glial immunoreactivity of pro-inflammatory cytokines in the hippocampus [27]. However, whether or to what extent this prolonged neuroinflammation would disturb hippocampal LTP formation and/or white matter function as reflected by CAP changes are still unknown. Thus in the current study, we used adult rats with mature white matter [16] to explore the effects of endotoxemia on functions of both hippocampus and white matter tracts after 3 daily systemic LPS injections. We examined these effects with two different measures: immediately following the last injection, and 3 days after the last injection, a cut off already associated with prolonged neuroinflammation [27, 28].

## Methods

### Animals

All animal experimental procedures followed the regulations of the ethics committee of the International Association for the Study of Pain [29]. Fifty-six adult male Sprague–Dawley rats (195–250 g) were provided by the Hunan SLAC Laboratory Animal Co. The rats were housed in isolated cages in a temperature-controlled (25–28 °C) and specific pathogen free room on a 12-h light/dark cycle with free access to food and water. All animals were randomly divided into LPS (*n* = 28) and saline peritoneal injection (*n* = 28) groups. We collected data at two time points: immediate following the last LPS or saline injections, and 3 days after cessation of both injections, which are referred to as day 3 and day 6 afterword. Animals in both groups were assigned into subgroups for electrophysiological recording of LTP (*n* = 8 in each group; 4 at day 3 and 4 at day 6) or CAPs (*n* = 8 in each group; 4 at day 3 and 4 at day 6), for Western blot examination of β-APP density (*n* = 6 in each group; 3 at day 3 and 3 at day 6) in the CC tissues, and for immunostaing of ion binding adaptor one (Iba-1; *n* = 6 in each group; three in day 3 and 3 in day 6) followed by proportional area measurement and analysis.

### Intraperitoneal injection (i.P.) of Lipopolysaccharide (LPS) and saline

LPS (*Escherichia coli*, L2880, Sigma, St Louise, MO) was prepared with sterilized saline at 1 mg/ml and stored at -20 °C. Animals in LPS group were intraperitoneally injected with 1 mg/kg LPS for 3 days; control animals received corresponding injections of saline. Half of the animals in each group were euthanized at day 3, and the rest were executed at day 6. The injections were made in the late evening and experiments started the next morning, and the peak of LPS toxemia may be reached 2 h after *i.p.* injection [9] with circulating cytokines peaking at 4 to 6 h after *i.p.* injection [27, 30].

### Electrophysiology

Animals were deeply anesthetized with isoflurane and decapitated. Brains were quickly removed and placed into an ice-cold (4 °C) oxygenated artificial cerebrospinal fluid (ACSF). Coronal brain slices primarily containing the CC and/or hippocampus were cut at 500 μm in cold oxygenated ACSF. Slices were incubated and oxygenated at room temperature (RT, 22–25 °C) for an hour before recording.

**Fig. 1:**
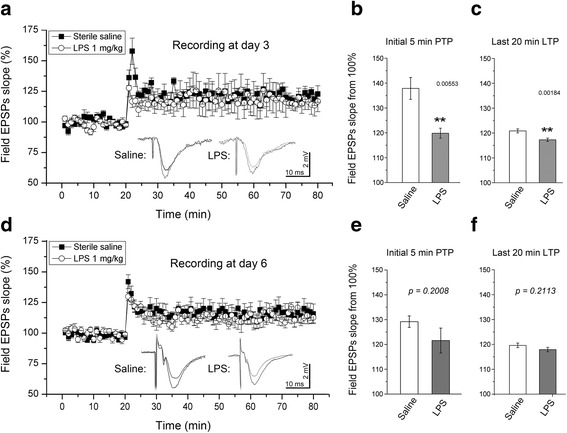
LTP recorded from hippocampal CA1 area and statistical comparison between two groups. **a**, LTP like responses were evoked in both groups at day 3 but post-tetanic potentiation (PTP) was obviously distorted in the systemic LPS group. **b**, statistical comparison of initial 5-min fEPSP values between two groups (*n* = 4 in each group) displayed a significant decrement of PTP in the LPS group (*p* < 0.01, **). **c**, statistical analysis of last 20-min fEPSP values between two groups exhibited a significant decline of LTP in the LPS group (*p* < 0.01). **d**, LTP like responses were elicited in both groups (*n* = 4 in each group) at day 6 --- 3-days after terminating LPS injections. **e**, statistical comparison of initial 5-min fEPSP values between two groups did not show significant difference in terms of PTP (*p* = 0.2008). F, statistical analysis of last 20-min fEPSP values between two groups neither show any significant difference in terms of LTP (*p* = 0.2113)

For hippocampus LTP recording, a bipolar Tungsten stimulating electrode (FHC, Bowdoin, ME) was placed on the Schaffer-collaterals and a glass microelectrode (2 ~ 5 MΩ) was placed onto the CA1 dendrite layer. The initial slope of field excitatory postsynaptic potentials (fEPSP) was recorded with 0.05 Hz stimuli for ~10–20 min if it is stable, which are averaged and multiplied 100% to be a baseline (Fig. 1a). Then, tetanic stimuli at 100 Hz was applied for a second twice to evoke LTP, followed by 0.05 Hz stimuli to record fEPSP for ~60 to 70 min. The slope values of fEPSP immediately after tetanic stimuli was recorded and plotted as PTP (we divided first 5 min of recording as hypothetical PTP values in statistical analysis). Afterword, recording of fEPSP slop values was continued for at least an hour, we consider LTP has been recorded if finally (we take last 20 min of recording as assumed LTP values in statistical process) the slop values are still significantly higher against the baseline (Fig. 1a).

For CC fiber CAP recording, the bipolar stimulating electrode was positioned on the corona radiate initially and then moved medially towards the other side. The glass microelectrode was placed on the CC fibers 1.5 to 1.7 mm away from the stimulating site. CAPs were evoked by a serial of incremental intensities (0.1 to 0.5 mA in 0.05 mA increments, 40 μs in duration, at 0.2 Hz) to build-up an input output curve (I/O). Area Under Curve (AUC) was used to measure extent of I/O curve downshifted.

The fEPSPs and CAPs were amplified with a Dagan EX4–400 amplifier (Dagan Corp., Minneapolis, MN) connected to an AXOPACH 1D (Axon Instruments Inc., Union City, CA), digitized at 5 kHz with a Digidata 1440A interface (Axon) and recorded on a Dell computer with pCLAMP 10.1 software (Axon).

### Western blot

The rats were euthanized with isoflurane and brain slices were prepared as the same way as described in the *Electrophysiology* section. The CC was dissected from coronal brain slices in cold ACSF under a dissecting microscope and quickly transferred into Tissue Extraction Reagent 1 (FNN0071, Invitrogen, Camarillo, CA) with 1:1000 protease inhibitor (P-2714, Sigma). Protein homogenate concentrations were determined by bicinchoninic acid assay (BCA assay). After centrifugation, the supernatant was subjected to sodium dodecyl sulfate-polyamide gel electrophoresis and then the protein was transferred to a PVDF membrane. The membranes were then blocked with 5% non-fat milk and incubated with rabbit anti-β-APP antibody (1:500; Abcam USA), followed by HRP-conjugated secondary antibody (1:5000; Jackson Immuno Inc., West Grove, PA). Mouse anti-β-actin (1:10,000; Sigma) was used for the gel loading control. Protein bands were detected using enhanced chemiluminescence and developed with autoradiography film. Image J (NIH, Bethesda, MD) software was used to quantify the band densities and protein densities of β-APP values were normalized by corresponding β-actin in each sample.

### Immunohistochemistry and proportional area measurement

The rats from both groups at both time-points were euthanized with isoflurane and perfused with saline and followed by 4% paraformaldehyde, then brains were removed and cryoprotected by gradient sucrose. Coronal frozen sections were cut at 10 μm thickness and mounted immediately. Sections were blocked with 10% normal goat serum followed by rabbit anti-ion binding adaptor-1 (Iba-1; 1:300; Wako USA) at RT overnight. Alexa Fluor 488 conjugated anti-rabbit (1:200, Molecular Probes, CA) was used for Iba-1 visualization. Control sections were blocked and stained with Alexa Fluor only. All sections were mounted using Vectashield with DAPI (Vector Labs, Burlingame, CA) and imaged with a Nikon-800 microscope. Morphological criteria for microglial activation status are well documented: ramified cells indicate a resting state, hyper-ramified to amoeboid cells represent a transformation from a resting to a reactive state, and non-ramified hypertrophic microglia represent a phagocytic state [31–33]. Proportional area measurement was applied to measure these morphological changes, which was acquired through 20X objective lens and measurement was performed by using Image J (NIH, Bethesda, MD). Selected area above threshold represented Iba-1 positive cells; while, base area was actually the rectangle of the 20X images excluding area of lateral ventricle, if any. The term of positive area in following context will mean selected positive area divided by base area.

### Statistical analysis

Statistical analysis was performed using GraphPad Prism software (GraphaPad Prism, La Jolla, CA). The data normality was evaluated using Kolmogorov-Smirnov test. Student’s *t*-test was used to determine significance of differences between LPS and saline injection groups for fEPSP values and β-APP densities. One-way ANOVA analysis of area under curve (AUC) was applied to reveal difference of I/O curves between LPS and saline injection groups. One-way ANOVA was also used to analyze proportional area changes among saline control, LPS injected cases at day 3 and at day 6. Significance was ascribed for *p* < 0.05. Level of significance are indicated by the number of symbols, *, significant, *p* < 0.05, **, very significant, *p* < 0.01, ***, extremely significant, *p* < 0.001.

## Results

**Fig. 2:**
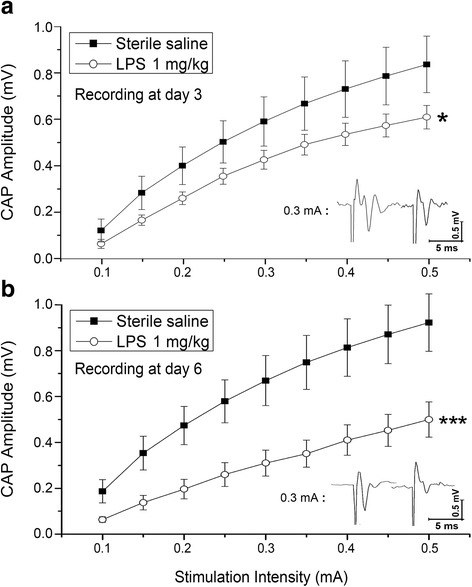
I/O curves from the CC nerve fibers CAP and analyzed with one-way ANOVA of area under curve (AUC) between two groups. **a**, I/O curves from the LPS (*n* = 4) group exhibited a significant downshift of the I/O curve at the day 3 against to the saline group (*n* = 4; *p* < 0.05, *). **b**, at the day 6 --- 3-day after cessation of saline (*n* = 4) and LPS (*n* = 4) injections, highly significant downshift of I/O curve in the LPS cases than in the saline injected one was revealed by one-way ANOVA AUC (*p* < 0.001, ***). This implies a prolonged injury during 3 days after the LPS injection was terminated

**Fig. 3:**
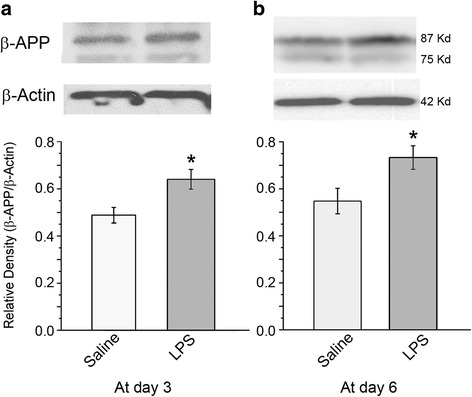
Western blot detection of β-APP changes in the CC and statistical comparison between groups. **a**, LPS (*n* = 3) injection induced an accumulation of β-APP in the CC tissues: after normalization by β-actin, the density of β-APP was significantly higher than that in saline (*n* = 3) group (*p* < 0.05, * by *t*-test). **b**, β-APP level in the CC from the rats 3 days after stopping LPS injections (*n* = 3) was still significantly higher than that in saline injection group (*n* = 3; *p* < 0.05, by *t*-test), suggesting a continued malfunction of axon fast transport

**Fig. 4:**
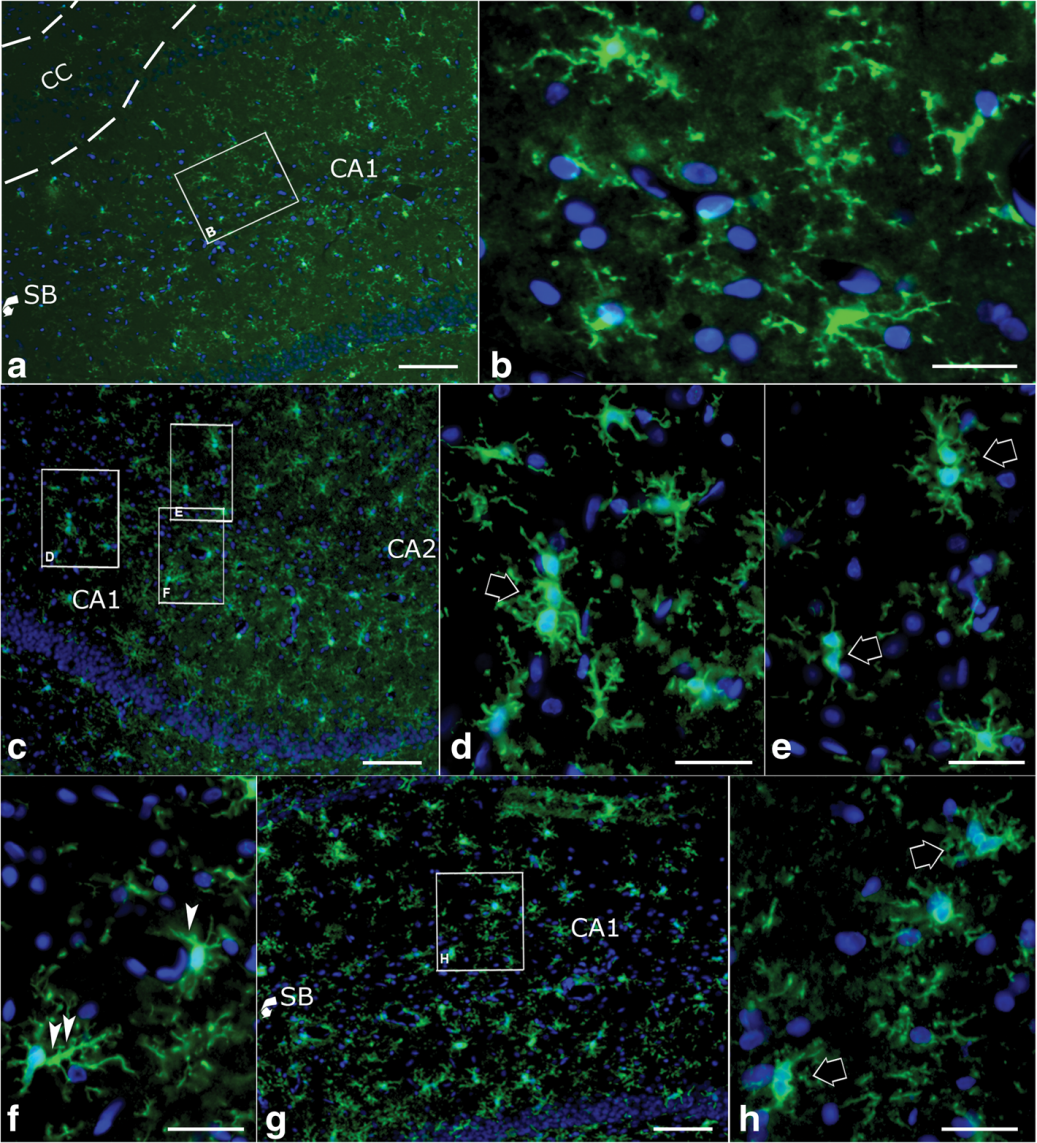
Microglia observed in hippocampus CA1 region from the saline and LPS injection groups. **a**, coronal section containing parts of the CA1 and CC regions from saline injected rats (*n* = 3), immunostained with anti-Iba-1 serum. **b**, high magnification of the framed area in the CA1 region from A, showing ramified microglia in this area that are probably in the resting state. **c**, representative image of CA1 region abutted by CA2 on a coronal section following LPS injections (*n* = 3) at the day 3, immunostained by anti-Iba-1 serum. **d**-**f**, high amplification images from the framed areas in **c**, showing that many Iba-1 labeled hyper-ramified cells with hypertrophic somata are clustered together (**d** and **e**, wide, open arrows), and some Iba-1 positive cells manifest hypertrophic pseudopodia either (F, small arrowheads). G, CA1 region near and above the SB from LPS injection group at the day 6 --- 3 days after cessation of the injections. **h**, high magnification of the framed area from the **g**, in which clustered Iba-1 positive cells with hypertrophic somata are still visualized, but clusters contain fewer somata (wide, open arrows). Scale bars in **a**, **c** and **g** are 150 μm, and in **b**, **d**, **e**, **f** and **h** are 25 μm. SB, subiculum

**Fig. 5:**
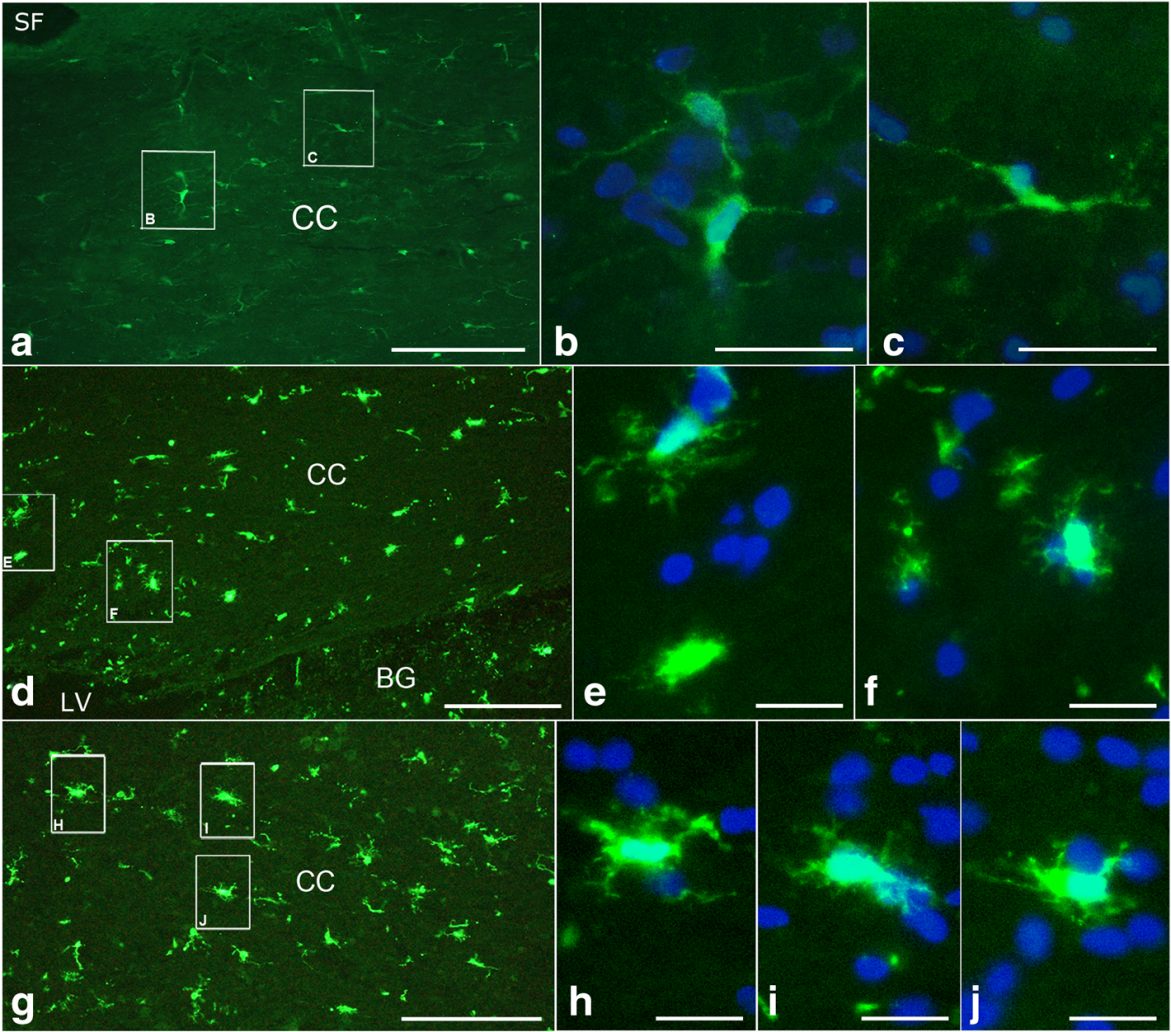
Microglia viewed in the CC region from the saline and LPS injection groups. **a**, anti-Iba-1 immunostained CC area from the saline injected animals (*n* = 3). **b** and **c**, high amplification of framed areas in **a**, showing resting microglia with thin somata and delicate pseudopodia in white matter. **d**, anti-Iba-1 immunostained CC area from LPS group at the day 3 (*n* = 3). **e** and **g**, high magnification images from the framed areas in **d**, showing Iba-1 stained cells with hypertrophic soamta and amoeboid appearance. **g**, anti-Iba-1 immnuostained CC region from LPS group (*n* = 3) at the day 6. **h** to **j**, high magnification of the framed areas in the **g**, showing that 3 days after termination of the LPS injection, microglia are still visualized as amoeboid appearance but with further hypertrophied somata. Scale bar in **a** is 150 μm. scale bars in **d** and **g** are 100 μm, in **b** and **C** are 25 μm, and in **e**, **f**, **h**, **i** and **j** are 20 μm. LV, lateral ventricle. BG, basal ganglion

**Fig. 6:**
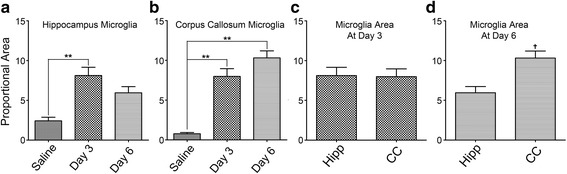
Microglia activation state evaluated by proportional area measurement of positive Iba-1 labeling. **a**, one-way ANOVA analysis of proportional area from hippocampus of saline and systemic LPS groups at both day 3 and day 6 showed that LPS injections significantly enhanced microglial area (*p* < 0.01, **) compared to saline control; further, microglial area decreased at day 6 compared to that at day 3. **b**, analysis of proportional area from CC of both groups at both day 3 and day 6 displayed that system LPS significantly enhanced microglial area (*p* < 0.01, by ANOVA) versus saline control; while, microglial area further increased at day 6 compared to that at day 3, and microglial area was still significantly higher than that in the saline group (*p* < 0.01, by ANOVA). **c**, comparison of proportional area after system LPS between hippocampus and CC at day 3 showed no significant difference (by *t*-test). **d**, however, proportional area in the CC post system LPS but 3-day after ending of system LPS was significantly higher than that values in the hippocampus (*p* < 0.05, shown as ^**ϯ**^, by *t*-test)

1. LTP recording: The rats were weighed every day; comparing to saline injection group, no significant weight lost was found in LPS injected rats (*p* > 0.05). LTP was elicited from both groups at day 3 judging from significant higher of fEPSP slopes values against baseline. However, comparing the recorded slop values between two groups, LPS injection significantly decreased PTP at this time point (Fig. 1b; *p* < 0.01, by *t*-test); further, the last 20 min recording show that LTP was also significantly declined by LPS injection at this time (Fig. 1c; *p* < 0.01, by *t*-test). This indicates that acute system LPS impaired hippocampus function in terms of PTP and LTP.
  Then, 3-day after cessation of injection, we performed the same recording at day 6. Similarly, PTP and LTP were evoked in both saline and LPS groups (Fig. 1d). While, comparing the two groups, no significant different PTP values between groups was disclosed by student *t*-test (Fig. 1e). Neither significant difference of last 20-min fEPSP slopes between two groups was identified (Fig. 1f). This inferred that function of LTP generation by hippocampus neurons has recovered 3-day after system LPS was terminated.
2. CAP recording: system LPS significantly downshifted I/O curve against saline injection at day 3 (Fig. 2a; *p* < 0.05). This result suggests a functional impairment of white matter tract. While contrast to results of hippocampal PTP and LTP recording, 3-day after cessation of the injection, the LPS injected cases showed a further downward shift of corresponding I/O curve at day 6 (Fig. 2b). One-way ANOVA comparison of AUC between the saline and LPS injected groups showed an extremely significant difference (Fig. 2b, *p* < 0.001), implying a progressing injury to white matter tracts.
3. β-APP Western blot: the density of β-APP in CC tissues of the LPS group was significantly higher than that in the saline group (Fig. 3a; *p* < 0.05 by *t*-test) at day 3. Then, 3-day after termination of saline and LPS injections, β-APP Western blot displayed similar results at day 6 as seen at day 3: the normalized protein level of β-APP in the CC of the LPS group was significantly higher than that of the saline group (Fig. 3b; *p* < 0.05 by *t*-test).
4. Microglia immunostaining: in the hippocampus of the saline injection group, Iba-1 labeled microglia appeared to have regular somata and bushy ramified pseudopodia (Fig. 4a, b). However, cells with thinner somata and delicate ramified pseudopodia were observed in the CC of saline injected rats (Fig. 5a-c).
  In the hippocampus of the LPS group, clusters of microglia with hypertrophic somata and bushy pseudopodia were frequently observed, and the cluster commonly formed by 2 ~ 4 cells (Fig. 4c-e, opened arrows). Hyper-ramified hypertrophic pseudopodia were also repeatedly encountered (Fig. 4f, arrowheads). Microglia in the CC were also markedly activated (Fig. 5d-f) at day 3 but with rather different characteristics from those in the hippocampus. Most of these activated microglia displayed shorter bushy pseudopodia and hypertrophic somata, with a characteristic amoeboid appearance (Fig. 5e, f). Clustered microglia with hypertrophic somata as observed in the hippocampus (Fig. 4d, e) was not encountered in the CC.
  Three days after cessation of LPS injections, microglia in the hippocampus still manifested clustered hypertrophic somata and short hyper-ramified pseudopodia but clusters were generally composed of a couple of somata (Fig. 4g, h; open arrows). Similarly, microglia in the CC of LPS group at day 6 were still showing an activated feature (Fig. 5g-j), similar to that observed at day 3 (Fig. 5d-f) as amoeboid morphology.
5. Proportional area analysis: the area that represents Iba-1 positive somata and pseudopodia in both hippocampus (Fig. 6a) and CC (Fig. 6b) were significantly expanded (*p* < 0.01, ** in both regions) at day 3 compared to the saline control. This reflects robust microglia activation. On the other hand, comparison of Iba-1 positive area in hippocampus with that in CC showed no obvious difference (Fig. 6c, by *t*-test). However, at 3-day after termination of LPS injection, the Iba-1 positive area in the hippocampus markedly decreased (Fig. 6a, by one-way ANOVA); whereas, this area in the CC evidently increased and values of this area were still significantly higher (Fig. 6b, *p* < 0.01 by one-way ANOVA) comparing to the saline group. Further, Iba-1 positive area in the CC was significantly greater than that in the hippocampus (Fig. 6d, *p* < 0.05, shown as ^**ϯ**^ **,** by *t*-test) at day 6.

## Discussion

The present study demonstrated acute systemic LPS declined LTP generation but we did not find a prolonged deterioration of hippocampus LTP generation 3-day after cessation of LPS injection that should be in a period of prolonged neuroinflammation. Based on previous studies in rodents, systemic LPS induced prolonged neuroinflammation could still be detectable even 1 to 4 weeks after single or repeated LPS injections [27]. Also in human studies of endotoxin related encephalopathy, it was observed that neuropsychiatric symptoms could continue after termination of experimental endotoxemia [28, 34]. We also examined microglia activation in hippocampus because a previous study showed attenuation of LTP was associated with microglia activation [35]. Based on our LTP recording and proportional area measurement data, it seems that impairment of LTP generation really correlated with microglia activation states, reflected by that significant reduction of LTP and increment of proportional area at the day 3, and recovery of LTP generation and attenuation of proportional area at the day 6.

The major finding of our current study is a progressive injury in mature white matter that continues after systemic LPS has been ended. In humans, white matter comprises 60% of the brain volume [36]; therefore, the roles that white matter may play in endotoxin related encephalopathy should not be overlooked. Clinical MRI studies have demonstrated marked white matter injuries in SAE patients [10, 11]. However, previously, direct noxious effects of endotoxin on white matter had been only demonstrated in fetal animals exposed through maternal endotoxemia [14, 15]. But there are vast structural and cellular-molecular differences between fetal, new-born and mature white matter [16–19]. It is known that maturation of brain white matter tract starts about 2 weeks postnatal in mice and 3 weeks postnatal in rats, and that myelination is essentially complete around postnatal 6 week [16, 19]. Surveys of children’s brain development at ages from neonatal to 11 years-old by diffusion tensor MRI and myelin water faction/relaxation MRI demonstrated that robust myelination is occurring from ~2 to 5 years of age, when it reaches ~80% of levels observed in white matter neuroimaging in adults [17, 18]. These data imply that mechanisms of mature white matter injury may be different from that of developing white matter. Hence, we examined alterations of mature white matter function at two time-points: immediately following systemic LPS, and 3-days after ending systemic LPS injections - a time point when LPS should have been completely cleared from the brain vasculature [9].

In the present work we used 6–8 week old rats, an age when myelination of the white matter tracts should be virtually completed [16]. In these rats, we indeed demonstrated white matter malfunction after initial systemic LPS as reflected by a downward shift of I-O curves plotted by CC nerve fiber CAP and significant accumulation of β-APP in the CC tissues. Though the I-O curve change immediately following LPS injections was significant compared to the saline groups, the greater downshift observed at the 3-day after end of system LPS was highly significant, suggesting an injury that continued to progress after injections stopped. At this time point, free circulating LPS in the brain should have been completely cleared because the half-time for clearance of LPS from the brain vasculature is 259 min [9]. It was reported that the concentration of LPS in the brain parenchyma is only 0.025% of the circulating LPS following a single injection [9]. In a previous study, even though repeated LPS injections increased permeability of the blood brain barrier, the level of LPS in the brain parenchyma was still not significantly elevated above the levels seen at single injection cases [9]. Therefore, the progressive white matter injury observed in this work is probably not a direct effect of LPS, but an enduring, indirect effect of prior systemic LPS, such as microglia activation by LPS. Anyhow, the current study demonstrated that: (1) endotoxemia will also cause insults to mature or well myelinated nerve fibers in addition to developing white matter in young animals; (2) this injury is progressive and not just a response to acute endotoxemia. Two mechanisms can be considered to explain the white matter impairment. One is circulating pro-inflammatory cytokines [27, 37, 38] and the other is cytokines originating in the brain, secreted by microglia and/or perivascular macrophages [25, 27]. Our proportional area analysis showed that microglial area values at 3-day after termination of systemic LPS is even higher than that values at a time immediately after systemic LPS. This observation supports the second mechanism, and is in consistent to prolonged neuroinflammation story [27]. Namely, it is possible that brain derived pro-inflammatory cytokines from the resident microglia in the white matter may exert direct effects on adjacent nerve fibers even after systemic LPS injections have ceased. It is noteworthy that, in a clinical investigation of the relationship between delirium and white matter injury, it was shown that the duration of delirium correlated with the degree to which white matter integrity was corrupted; moreover, white matter disruption detected by MRI was found not only at the time of discharge but also 3 months later [13]. In addition, white matter disruption was also correlated with worse cognitive scores even 12 months after discharge [13].

## Conclusion

In summary, we found that both the hippocampus and the CC nerve fibers showed malfunction immediately following the systemic LPS, as demonstrated by a significant attenuation of hippocampal PTP and LTP, and by a significant decrement of CC nerve fibers CAP and accumulation of β-APP. The hippocampus appeared to be more sensitive to acute endotoxemia; however, prolonged LTP declination was not found. In contrast, the CC function might be initially lessened during systemic LPS and the injury seemed prolonged further when measured 3-days after cessation of systemic LPS. These results suggested SAE, manifested by delirium, might not be regarded as an acute reversible state. Sepsis induced a multifaceted syndrome with a multiple brain areas injuries. Some subclinical injuries to white matter tract were probably prolonged even after the endotaxemia and delirium had been controlled. Therefore, continued clinical efforts should be made to protect brain function rather than to merely control the major SAE symptoms.

## Abbreviations

ACSF: Artificial cerebrospinal fluid
CAP: Compound action potentials
CC: Corpus callosum
fEPSP: Field excitatory postsynaptic potentials
i.p: Intraperitoneal injection
LPS: Lipopolysaccharide
LTP: Long term potentiation
MRI: Magnetic resonance imaging
PTP: Post tetanic potentiation
SAE: Sepsis associated encephalopathy
β-APP: Beta amyloid precursor protein
